# Methane production by treating vinasses from hydrous ethanol using a modified UASB reactor

**DOI:** 10.1186/1754-6834-5-82

**Published:** 2012-11-21

**Authors:** Elda I España-Gamboa, Javier O Mijangos-Cortés, Galdy Hernández-Zárate, Jorge A Domínguez Maldonado, Liliana M Alzate-Gaviria

**Affiliations:** 1 Unidad de Energía Renovable, Centro de Investigación Científica de Yucatán A.C (CICY), Calle 43 No. 130 Col. Chuburná de Hidalgo, C.P. 97200, Mérida, Yucatán, Mexico; 2 Unidad de Recursos Naturales, CICY A.C, Mérida, Mexico; 3 Departamento de Ciencias Marinas, Instituto Tecnológico de Boca del Río, Km 12 Carretera Veracruz-Córdoba, Boca del Río, Veracruz, C.P. 94290, Mexico

**Keywords:** Methane yield, Modified UASB reactor, Vinasse from hydrous ethanol, 16S-rDNA genes amplification

## Abstract

**Background:**

A modified laboratory-scale upflow anaerobic sludge blanket (UASB) reactor was used to obtain methane by treating hydrous ethanol vinasse. Vinasses or stillage are waste materials with high organic loads, and a complex composition resulting from the process of alcohol distillation. They must initially be treated with anaerobic processes due to their high organic loads. Vinasses can be considered multipurpose waste for energy recovery and once treated they can be used in agriculture without the risk of polluting soil, underground water or crops. In this sense, treatment of vinasse combines the elimination of organic waste with the formation of methane. Biogas is considered as a promising renewable energy source. The aim of this study was to determine the optimum organic loading rate for operating a modified UASB reactor to treat vinasse generated in the production of hydrous ethanol from sugar cane molasses.

**Results:**

The study showed that chemical oxygen demand (COD) removal efficiency was 69% at an optimum organic loading rate (OLR) of 17.05 kg COD/m^3^-day, achieving a methane yield of 0.263 m^3^/kg COD_added_ and a biogas methane content of 84%. During this stage, effluent characterization presented lower values than the vinasse, except for potassium, sulfide and ammonia nitrogen. On the other hand, primers used to amplify the 16S-rDNA genes for the domains Archaea and Bacteria showed the presence of microorganisms which favor methane production at the optimum organic loading rate.

**Conclusions:**

The modified UASB reactor proposed in this study provided a successful treatment of the vinasse obtained from hydrous ethanol production.

Methanogen groups (Methanobacteriales and Methanosarcinales) detected by PCR during operational optimum OLR of the modified UASB reactor, favored methane production.

## Background

Worldwide ethanol production for fuel (hydrous and anhydrous ethanol), pharmaceutical use, industrial use and alcoholic beverages has increased in recent years, generating between 9 and 14 litres of wastewater known as vinasse for each litre of ethanol obtained. Vinasse has a pH between 3.5 and 5, a dark brown color and a high chemical oxygen demand (COD) which ranges between 50 and 150 g/L [1,2]. Vinasse has been reported to be used for irrigation and fertilization due to its high nutrient and organic matter content. Nevertheless, the presence of phytotoxic, antibacterial and recalcitrant compounds such as phenols, polyphenols and heavy metals has been observed to cause negative effects on microorganisms and plants in discharge areas [1,3]. It is therefore necessary to subject this waste to a conditioning treatment prior to its disposal in the environment [4].

Different technologies exist for treating vinasses [2,3]. The anaerobic sludge blanket reactor (UASB) is the most popular anaerobic digester; it has been used for the treatment of many types of industrial wastewaters (including the vinasses), due to its high treatment capacity compared with other systems [3]. The advantages it offers include low sludge production and conversion of over 50% of the COD to biogas, which is considered to be a renewable energy source [1]. The greatest methane production yield reported by studies on anaerobic treatment of different types of vinasses is 0.344 m^3^/kg COD_removed_[5-9] and the breakdown of organic matter has been observed to be performed by a microbial consortium of anaerobic bacteria and methanogenic archaea. In general terms, the domain Bacteria is mainly represented by the Firmicutes and Bacteroidetes phyla [10]. Methane production is performed by the domain Archaea, represented by the Methanococcales*,* Methanobacteriales*,* Methanomicrobiales and Methanosarcinales orders [10,11]. However, this microbial diversity can vary in terms of the operational characteristics of the reactor and the substrate employed.

The aim of this study was to determine the optimum organic loading rate for operating a modified upflow anaerobic sludge blanket (UASB) reactor to treat vinasse generated in the production of hydrous ethanol from sugar cane molasses.

## Results and discussion

### Vinasse characterization

The properties of the vinasse used in this study are shown in Table 1. Its composition can be seen to be acidic, with a high COD (121,000 mg/L) and sulfate (5,336 mg/L) content. Kumar *et al*. [8] report that in hydrous alcohol vinasse, the COD is found to be between 90,000 and 130,000 mg/L, whilst the sulfate content is between 6,000 and 6,500 mg/L. 

**Table 1:** Vinasse characterization

**Parameter**	Value^*^
pH	4
COD	121000
SO_4_^2-^	5336
S^-^	168
N_T_	1341
N-NH_3_	160
PO_4_^3-^	141
K^+^	7262
Ethanol	21007
Acetic Acid	2237
Propionic Acid	4304
Suspended solids	20273
Dissolved solids	45543

^*^ All values except pH are expressed in mg/L.

Ammonia nitrogen and sulfide exert inhibitory effects on anaerobic digestion and consequently affect methane yield. The literature reports wide ranges for ammonia nitrogen from 1,700 to 14,000 mg/L and between 30 and 250 mg/L for sulfide [12,13]. However, in the case of ammonia nitrogen, results have been reported to be beneficial for anaerobic digestion at concentrations of around 200 mg/L [12]. In this study, the ammonia nitrogen concentration of the vinasse was 160 mg/L and the sulfide concentration was 169 mg/L, meaning that the ammonia nitrogen value was found to be beneficial for anaerobic digestion, whilst sulfide had an inhibitory effect. Carboxylic acids such as acetic, propionic and butyric acids are substrates for the anaerobic digestion process. However, Parawira *et al*. [13] has demonstrated that values above 10,000 mg/L of total volatile fatty acids (VFAs) can also cause an inhibitory effect by reducing pH, which without sufficient buffering capacity inhibits the initiation of methane production. The vinasse in this study had a total VFA concentration of approximately 7,000 mg/L, which is approaching the inhibitory level, and therefore required the addition of NaHCO_3_ as a buffer to prevent a sharp drop in system pH.

### Reactor start-up

This study used granular inoculum from a UASB reactor operated to treat vinasse of banana waste, so, the start-up of the modified UASB reactor was subjected to an acclimatization period with an OLR of 0.34 kg COD/m^3^-day using 200 ml/day of Synthetic Wastewater (SW) during the first six days after inoculation, 17% CH_4_ and 72% COD removal was reached. On the seventh day, the loading rate was increased to 5.9 kg COD/m^3^-day (150 ml/day of hydrous ethanol vinasse) and biogas production of 2 L per day was obtained (38% of CH_4_ and 84% of COD removal). Finally, on the ninth day and with the same loading rate, biogas methane concentration was 58% and 97% COD removal.

Molina *et al*. [14] obtained a start-up time of 60 days in a hybrid reactor for treating wine vinasse, using flocculent sludge which were collected from two anaerobic digesters for processing wastewater from a sugar factory and the fiberboard production process as the inoculum. These authors used an initial organic loading rate of 0.5 kg COD/m^3^-day, which was increased to reach 5 kg COD/m^3^-day, and obtained removal of 98% of the COD and a biogas methane content of between 70 and 74%. Similarly, Gao *et al*. [15] obtained a start-up time of 40 days in a UASB reactor for the anaerobic treatment of vinasse from wine production under mesophilic conditions using flocculent sludge from the anaerobic treatment of residential wastewater as the inoculum. The UASB reactor was operated with an initial organic loading rate of 0.42 kg COD/m^3^-day, which was increased to reach 5.6 kg COD/m^3^-day, and obtained COD removal of 93.8% and a biogas methane content of 60%. The COD removal values obtained in this study agrees with the results obtained by Molina *et al*. [14] and Gao *et al.*[15] when they evaluated nearly the same OLR. Nevertheless, it is clear that start-up times were longer, attributed to the flocculent state of the inoculation sludge. The authors considered their start-up stage to be complete when granules could be distinguished in the reactor bed. A longer start-up time benefitted Molina *et al.*[14], who obtained a higher biogas methane concentration.

On the other hand, Wolmarans and de Villiers [16] studied the start-up period of a UASB reactor for the treatment of vinasse from sugar cane molasses using granular inoculum from a UASB operated to treat wastewater from a brewery. This process was stabilized in 7 days with an organic loading rate of 8 kg COD/m^3^-day and COD removal of over 90% was obtained. These results match the ones in this study, given that the use of granular inoculum increases methane production as a result of their high metabolic activity. This causes the process to reach higher yields in shorter time periods, thereby reducing start-up time. Another important factor is the fact that the inocula in both studies were previously obtained from anaerobic reactors for the treatment of vinasses. Vadlani and Ramachandran [17] showed that by using sludge from the anaerobic treatment of vinasses as the inoculum in the start-up of a UASB reactor, the time can be reduced by up to 40% compared to anaerobic sludge from residential wastewater treatment, given that specific methanogenic activity is greater in sludge from vinasse treatment.

### Modified UASB performance

**Figure 1:**
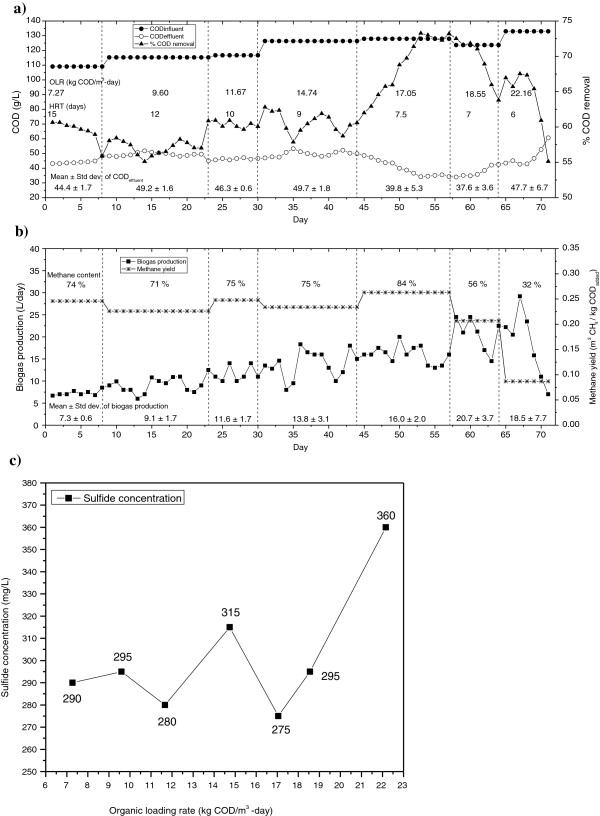
Evaluation of performance.

The organic loading rates, hydraulic retention times (HRT), COD influent, COD effluent and % COD removal, are shown in Figure 1a. During this study the vinasse introduced was not subjected to any dilution process. The optimum loading rate selected in this study was 17.05 kg COD/m^3^-day, which corresponded to the highest biogas methane content of 84%, methane yield of 0.263 m^3^ CH_4_/ kg COD_added_ and 69% COD removal (the level of COD removal increased in this stage). Finally, the system collapsed at an OLR of 22.16 kg COD/m^3^-day (Figure 1a and b). 

The theoretical methane yield expressed in cubic meters per kilograms of COD consumed should be 0.35, assuming that all of the incoming COD is transformed into methane, and considering that the biomass growth and cell maintenance is null [18]. The methane yield value obtained in the present work (0.263 m^3^ CH_4_/ kg COD_added_) could be explained by the presence of significant sulfate concentrations (5,336 mg/L) [3]. The reduction in reactor performance is attributed to the reduction of the sulfate present in the vinasse to sulfide. Inhibitory sulfide levels reported in the literature were in the range of 100–800 mg/L dissolved sulfide or approximately 50–400 mg/L undissociated H_2_S. The latter it can diffuse into the cell membrane. Once inside the cytoplasm, H_2_S may be inhibitory by denaturing native proteins through the formation of sulfide and disulfide cross-links between polypeptide chains, interfering with the various coenzyme sulfide linkages, and interfering with the assimilatory metabolism of sulfur and therefore reduces COD removal and methane yield [19]. As can be seen in Figure 1b and c, the methane yield increases when the sulfide concentration is reduced. It is important to highlight that the sulfide was present in the modified UASB reactor throughout the experimental period, demonstrating a significant negative effect on methane yield for concentrations of 360 mg/L.

Table 2 compares the modified UASB reactor in this study with the results of other authors. This study can be placed in the mid-to-high range within the literature with respect to methane yield of 0.263 m^3^/kg COD_added_. Molina *et al*. [14], who worked with winery effluent (less complex vinasses), obtained a high methane yield (0.33 m^3^ CH_4_/ kg COD_added_), due to the fact that these authors used an USBF reactor (UASB + anaerobic filter), developing granular biomass with suitable specific methanogenic activity and very good settling characteristics. An adequate biogas quality was also obtained (70–74% CH_4_). Likewise, the works which presented high COD removal performance are found to be related to the treatment of vinasses originating from the production of alcoholic beverages such as beer and wine. COD values in the literature for brewery effluents are found to range between 1,000 – 6,000 mg/L and between 26,000 – 50,200 mg/L for winery wastewaters [3,20]. These values are lower than for the vinasses used in this study (121,000 mg/L). Furthermore, vinasses obtained from hydrous ethanol production from sugar cane molasses present high sulfate, potassium and iron concentrations compared to vinasses from alcoholic beverages [3,7]. This leads us to assume that the low percentage COD removal achieved in this study is a result of the high complexity of the vinasse used, which can be corroborated with other similar studies where the substrate used was hydrous alcohol vinasse [6,21-23]. Likewise, the HRT in this study is found amongst the higher values obtained by previous studies, due to the fact that the vinasse was fed undiluted into the modified UASB reactor, which causes a high organic loading rate at low flow rates (L/day), thereby saving water resources which permit a reduction in the discharge volumes of the modified UASB reactor. 

**Table 2:** Comparison of performance parameters of UASB by different types of

**Vinasses**	**OLR kg COD/m**^**3**^**-day**	**HRT days**	**COD**_**removed**_**%**	**CH**_**4**_**%**	**M.Y. m**^**3**^**CH**_**4**_**/kg COD**_**added**_	**Reference**
Vinasse from hydrous alcohol distillery plant, using UASB (laboratory scale)	24.00	4.0	75	58	0.217	[21]
Cane molasses vinasse from hydrous alcohol distillery plant diluted ten-fold, using UASB (pilot scale)	19.00	0.5	40	na	0.210	[6]
Cane molasses hydrous alcohol stillage, using UASB (laboratory scale)	14.49	9.0	65	na	0.055	[22]
Diluted brewery wastewater, using UASB (laboratory scale)	1.53	0.75	91	67	0.209	[7]
Winery effluent treatment in an anaerobic hybrid USBF (pilot scale)	12.00	7.0	96	74	0.330	[14]
Wheat straw vinasse, using a UASB (laboratory scale)	17.10	2.0	76	64	0.155	[23]
Vinasse from hydrous ethanol distillation, using a modified UASB reactor (laboratory scale)	17.05	7.5	69	84	0.263	This Study

na: data not available.

### Characterization of the effluent obtained at the optimum organic loading rate

Table 3 shows the physicochemical characterization of the influent and the effluent obtained after anaerobic treatment at the optimum organic loading rate. 

**Table 3:** Physicochemical characterization of influent and effluent at the optimum organic loading rate

**Parameter**	Influent^*^	Effluent^*^	Removal percentage^**^
pH	4.51	7.22	-
COD	125600	39810	69
SO_4_^2-^	5433	0	100
S^-^	175	275	-
N_T_	1377	1160	16
N-NH_3_	113	230	-
N_organic_	1263	930	26
PO_4_^3-^	147	117	21
K^+^	6706	6838	-
Ethanol	19901	232	99
Acetic acid	2697	331	88
Propionic acid	3009	2283	24
Butyric acid	0	0	-

^*^ All values except pH are in mg/L.

^**^ (−): Not applicable.

Potassium (K^+^) was not removed; this corroborates the finding that anaerobic digestion does not favor elimination of this element. Information on the effect of application of vinasses on physical properties of soil is limited. However, application of wastewaters with high potassium levels has been found to increase the overall level of soil fertility, with the exception of alkaline effluents which can dissolve soil organic carbon [24].

In anaerobic reactors, sulfate is reduced to sulfide by the sulfate reducing bacteria (SRB). Sulfate reduction is performed by two major groups of SRB including incomplete oxidizers, which reduce compounds such as lactate to acetate and CO_2_, and complete oxidizers, which completely convert acetate to CO_2_ and HCO_3_[19]. Kumar *et al*. [8] showed that once removal of 80% of the sulfate present in hydrous ethanol vinasse was obtained, the sulfide concentration rose to 400 mg/L, which inhibited the microorganisms and led to a reduction in methane yield. Two stage of inhibition exist for methanogenic bacteria because of the sulfate reduction; primary inhibition is due to competition for common organic and inorganic substrates from SRB, which suppresses methane production, the sequence of the affinity of SRB for reduced substrates is Hydrogen > propionate > other organic electron donors. Because of the variety in substrate utilization exhibited by SRB, they compete with several different types of microorganisms involved in anaerobic digestion. Secondary inhibition results from the toxicity of sulfide, the inhibitory sulfide levels reported in the literature were in the range of 100–800 mg/L dissolved sulfide or approximately 50–400 mg/L undissociated H_2_S, Fermentative microorganisms which are responsible for the breakdown of monomers into smaller products were less affected by sulfide toxicity than SRB, or Methane producing bacteria; toxicity thresholds for acetogens were comparable with those of the SRB. Sulfur is a required nutrient for methanogens. It has been shown that the sulfur content of methanogens was higher than in other groups of microorganisms generally found in anaerobic systems. The optimal level of sulfur reported in the literature varies from 1 to 25 mg/L. The levels reported in the literature for inhibition of Methane producing bacteria also vary, with IC50 values of 50–125 mg H_2_S/L at pH 7–8 for suspended sludge and 250 mg H_2_S/L and 90 mg H_2_S/L at pH 6.4–7.2 and pH 7.8–8.0, respectively [19]. In this study, sulfate removal was 100%; the quantity of sulfide in the effluent was 275 mg/L at the optimum organic loading rate.

Harada *et al*. [6] obtained a maximum concentration of 300 mg/L of acetic acid, 1,200 mg/L of propionic acid, a methane yield of 0.21 m^3^ CH_4_/kg COD_added_ and COD removal of 40% with a UASB reactor operated at 19 kg COD/m^3^-day employing hydrous ethanol vinasse. Likewise, during treatment of whiskey distillery wastewater at 18 kg COD/m^3^-day, Goodwin and Stuart [5] found that biogas production was 6 L/day (50% less than at the previous loading rate), obtaining acetic and propionic acid levels of 900 mg/L and 6,000 mg/L respectively and COD removal of 50%. Ethanol and acetic acid removal are undoubtedly greater than propionic acid removal, given that these two compounds have a methane conversion rate of 3.56 and 3.92 mmol CH_4_/g VS-day respectively. In contrast, the rate for propionic acid is 0.55 mmol CH_4_/g VS-day [25]. This suggests that propionic acid is one of the VFAs which microorganisms have difficulty breaking down during anaerobic digestion.

In this study, trough out the optimum organic loading rate (45 to 55 days) the VFA were acetic and propionic acids; but, since the begging in the vinasse the acetic (2,697 mg/L) and propionic (3,009 mg/L) acids were present. In the VFA profile of the effluent, the acetic acid changed from 0 mg/L to 331 mg/L, while the propionic acid the values began at 1,429 mg/L and ended with 2,283 mg/L. Although, the acetic acid level was similar to that obtained by Harada *et al*. [6], the methane yield (0.263 m^3^ CH_4_/kg COD_added_) and COD removal (69%) were higher, this suggests that the greater quantity of acetic acid present was transformed into methane.

### Microbial identification

In previous research, the microorganisms found in anaerobic reactors are wide-ranging and vary depending on the substrate and operating conditions employed in the bioreactors. Table 4 shows the amplification results of the 16S-rDNA genes of the domains Bacteria and Archaea obtained at the optimum organic loading rate in this study. 

**Table 4:** Microbial groups evaluated by 16S-rDNA amplification at the optimum organic loading rate in the modified UASB reactor

**Domain**	**Group**	Amplicon*
	Archaea	+
Archaea	Methanogens	+
	Methanobacteriales	+
	Methanosarcinales	+
	*α*-Proteobacteria	+
	*β*-Proteobacteria	+
Bacteria	*γ*-Proteobacteria	+
	*δ*-Proteobacteria	+
	High GC Gram-positive Bacteria	-
	Low GC Gram-positive Bacteria	+
	*Bacillus*	+
	Sulfate Reducing Bacteria (SRB)	+
	*Clostridium*	+

*Presence (+) or absence (−) of the specific amplification product.

The sulfate concentration was above 5,000 mg/L in the hydrous ethanol vinasse in this study, the presence of species from Methanobacteriales and Methanosarcinales orders in the domain Archaea were present in the optimum OLR. The *Methanobacterium* and *Methanosaeta* species belong to these orders respectively, which suggests that they were present in the bioreactor. This result was similar to the one obtained by Sarti *et al*. [26], who performed the characterization of methanogenic archaea in an anaerobic reactor under mesophilic conditions for the treatment of wastewater rich in sulfates (between 1,000 and 3,000 mg/L) with a COD/SO_4_^2-^ ratio of 1.8 and 1.5. Considering the operational condition of 1,000 and 2,000 mg SO_4_^2-^/L, it was observed the presence of methanogenic archaea (99% of similarity with *Methanosaeta* spp.). At concentration of 3,000 mg SO_4_^2-^/L the methanogenesis was inhibited and methanogenic organisms were not detected in the clone library. Likewise, Oude *et al*. [11] indicated that *Methanosaeta* spp. were the dominant acetate degraders, and *Methanobacterium* spp. the dominant hydrogen- and formate-consuming methanogens in the treatment of wastewater with a COD/SO_4_^2-^ ratio of 9.5, while *Desulfobulbus* spp. and *Syntrophobacter* spp. were important for propionate degradation (sulfate reduction).

The bacterial groups found in this study, were similar to other bacterial identification research in anaerobic digesters [10,11,26], where the group of Gram-positive bacteria with low GC (included in the Firmicutes phylum) is composed of a large number of bacterial genera including *Bacillus, Clostridium, Enterococcus, Lactobacillus* and *Lactococcus*, which perform the stages of hydrolysis and acidogenesis [10]. The detection of the δ-Proteobateria subclass in this reactor suggests the presence of bacterial genera capable of using sulfate as an inorganic substrate, which achieve removal of over 95% of initial sulfates in the hydrous ethanol vinasse [11,26]. It is important to highlight that the absence of Gram-positive Bacteria with a high GC content, a well-known group due to its inclusion of different pathogenic species (independently of the γ-Proteobacteria subclass), could benefit the use of this type of effluent in combined fertilization and irrigation systems.

## Conclusions

The modified UASB reactor proposed in this study provided a successful treatment of the vinasse obtained from hydrous ethanol production. The optimum organic loading rate found experimentally was 17.05 kg COD/m^3^-day corresponding to a HRT of 7.5 days and a methane yield of 0.263 m^3^/kg COD_added_. During operational optimum organic loading of the modified UASB reactor, the group of methanogenic archaea belonging to the Methanobacteriales and Methanosarcinales orders favored methane production.

## Methods

### Reactor design

**Figure 2:**
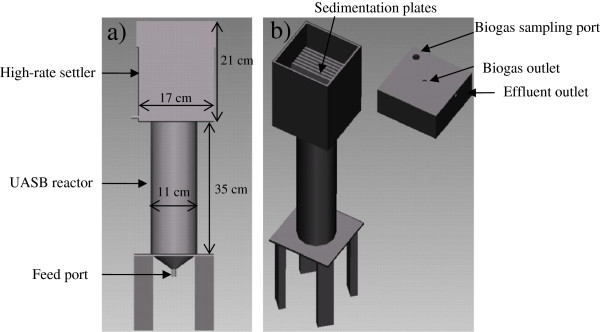
Modified UASB.

A modified UASB reactor was designed and built from acrylic with an operational volume of 3 L. The modification consisted of equipping the top part with a high-rate settler with plates inclined at 45 degrees (Figure 2), designed to retain volatile suspended solids and subsequently recirculate them to the reactor sludge blanket. 

### Chemical analyses

COD, total nitrogen (N_T_), ammonia nitrogen (N-NH_3_), phosphate (PO_4_^3-^), sulfate (SO_4_^2-^) and sulfide (S^2-^) content, both in the vinasse obtained from hydrous ethanol production and in the reactor effluent, were determined via colorimetric methods (Hach Company DR-890), whilst the pH and potassium content (K^+^) were determined in accordance with American Public Health Association [27].

The concentration of VFAs and ethanol were determined by gas chromatography in a Clarus 500-Perkin Elmer equipped with flame ionization detector (FID), using the EC^TM^ – 1000 column (30 m long, 0.32 mm internal diameter and 0.25 μm film thickness), nitrogen as the carrier gas and temperatures of 240, 160 and 250°C for the injector, oven and detector respectively.

The volume of biogas produced was measured every 24 hours with an acidified saline solution gasometer in accordance with the procedure reported by Poggi *et al*. [28] and biogas methane concentration was determined once again with the Clarus 500-Perkin Elmer with the thermal conductivity detector (TCD), a Molesieve column (30 m long, 0.53 mm internal diameter and 0.25 μm film thickness), nitrogen as the carrier gas and temperatures of 75, 30 and 200°C for the injector, oven and detector respectively.

### Reactor inoculation and start-up

The reactor was inoculated with 2.5 L of granulated sludge from a UASB reactor operated to treat vinasse obtained from the production of ethanol from banana waste. The substrate source consisted of 1 L of SW in accordance with the composition described previously [29].

The modified UASB was subsequently operated under mesophilic conditions (30 ± 5°C) and fed semi-continuously with SW for 6 days. A pH of close to 7.0 was maintained during this stage using sodium bicarbonate (NaHCO_3_) as a buffer. Finally, once biogas production was observed, the reactor was fed with 150 ml of hydrous ethanol vinasse from sugar cane molasses per day and start-up was deemed successful on obtaining biogas methane content and COD removal values greater than or equal to 55% and 95% respectively.

### Optimum organic loading rate evaluation

The performance of the modified UASB reactor was evaluated for 7 organic loading rates: 7.27, 9.60, 11.67, 14.74, 17.05, 18.55, 22.16 kg COD/m^3^-day, with a view to finding the optimum rate. These loading rates were obtained based on an initial flow rate of 200 ml/day of undiluted vinasse and progressive increases of 50 ml/day.

### Identification of bacterial groups and methanogenic archaea

Once the optimum organic loading rate was reached for the modified UASB reactor, sludge samples were collected aseptically and stored at −70°C in a deep freezer. Total DNA was extracted directly from 250 mg of moist sludge using a PowerSoil^TM^ DNA Isolation Kit (Mo-Bio Laboratories, CA), in accordance with the manufacturer’s instructions. Fragments of the 16S-rDNA genes were amplified by PCR using a universal 16Sf primer and one of the 9 previously described specific primers for each bacterial group or genus examined (α-, β-, δ-, and γ-Proteobacteria subclasses, Gram-positive bacteria with high and low GC content, sulfate-reducing bacteria (SRB), and for the genera *Bacillus* and *Clostridium*) [30]. In addition, specific primers based on previously described sequences were used to amplify the 16S-rDNA genes of methanogenic archaea belonging to the Methanobacteriales and Methanosarcinales orders [31]. 16S rRNA genes amplification was performed under the following conditions: 6 min of initial denaturation at 95°C followed by 25 amplification cycles at 95°C for 1 min, specific annealing temperatures for 1 min and 72°C for 2 min. An extra extension step of 10 min at 72°C was added after completion of the 25 cycles. The amplified products were analyzed on 1.2% agarose gels in 1X TBE buffer. The gels were stained with ethidium bromide (25 mg/ml) and photographed under UV light (302 nm, LMS 20E, UVP Inc., Upland, CA).

### Data analysis

Experimental data were collected and further processed with the Statistica 7 software (Statsoft®); the descriptive data was obtained and shown in the corresponding figures through the mean and standard deviation descriptors.

## Abbreviations

UASB: Upflow anaerobic sludge blanket; COD: Chemical oxygen demand; OLR: Optimum organic loading rate; VFAs: Volatile fatty acids; HRT: Hydraulic retention time; M.Y.: Methane yield; VS: Volatile solids; N_T_: Total nitrogen; N-NH_3_: Ammonia nitrogen; PO_4_^3-^: Phosphate; SO_4_^2-^: Sulfate; S^2-^: Sulfide; FID: Flame ionization detector; TCD: Thermal conductivity detector; SW: Synthetic wastewater; SRB: Sulfate-reducing bacteria.

## Competing interests

The authors’ declare that they have no competing interests.

## Authors’ contributions

EIEG performed the experiments presented herein and drafted the manuscript. JOMC assisted in experimental design and in drafting the manuscript. GHZ supervised the molecular analysis of microbial communities by PCR and assisted in drafting the manuscript. JADM developed the carboxylic acids analysis. LMAG conceived of the study, supervised the work, designed the modified UASB reactor and drafted the manuscript. All authors participated in the experimental design, evaluation of the data, read and approved the final manuscript.
